# Genetic variations and plasma levels of gelatinase A (matrix metalloproteinase-2) and gelatinase B (matrix metalloproteinase-9) in proliferative diabetic retinopathy

**Published:** 2008-06-14

**Authors:** Michal Beránek, Petr Kolar, Svatava Tschoplova, Katerina Kankova, Anna Vasku

**Affiliations:** 1 Department of Pathophysiology, Faculty of Medicine, Masaryk University, Brno, Czech Republic; 2 Department of Ophthalmology, University Hospital Brno-Bohunice, Brno, Czech Republic

## Abstract

**Purpose:**

Matrix metalloproteinases (MMPs) are postulated to be involved in the development of retinal angiogenesis through the regulation of extracellular matrix. The objective of the present study was to test for a possible association of five single nucleotide polymorphisms (SNPs) in the *MMP-2* gene and two polymorphisms in the *MMP-9* gene with proliferative diabetic retinopathy (PDR) and to determine their plasma levels.

**Methods:**

The study comprised 490 Caucasian participants, who were divided into three groups: diabetics with PDR, diabetics without PDR, and nondiabetics. Genotypes were detected by polymerase chain reactions followed by restriction analyses with specific endonucleases and their frequencies determined. Plasma levels of MMP-2 and MMP-9 proteins were analyzed by ELISA.

**Results:**

Neither *MMP-2* SNPs nor *MMP-9* SNPs revealed significant association with PDR in single-locus comparisons; similarly, *MMP-2* haplotype frequencies did not differ notably between groups, although the C-allele of the −1306C/T polymorphism and the C-allele containing haplotype (CGCG) in *MMP-2* exhibited marginally significant association with PDR in males (p<0.05, p_corr_=NS). Both MMP-2 and MMP-9 plasma levels showed statistically significant differences among the studied groups (p<0.001 and p=0.001, respectively) with highest levels in the PDR group. MMP-2 plasma levels were markedly higher in carriers of either the −1306CC and −1306CT genotypes and (p=0.009) or CGCG haplotype (p=0.043).

**Conclusions:**

These findings indicate that genotype- and haplotype-specific effects on *MMP-2* expression corresponding with its plasma levels may contribute to the susceptibility to PDR.

## Introduction

Proliferative diabetic retinopathy (PDR) is characterized by active angiogenesis in the retina and the formation of fibrovascular tissue at the vitreoretinal interface [1]. Angiogenesis involves migration, proliferation, differentiation, and adhesion of cells and is influenced by the surrounding extracellular matrix (ECM). These processes require regulation and local production of angiogenic factors [2,3] as well as synthesis of ECM components necessary for the anchorage of migrating endothelial and other cells, such as retinal pigment epithelium, glial cells, and fibroblasts [4,5]. Degradation of ECM proteins is exerted by matrix metalloproteinases (MMPs), whose activity is, in turn, regulated by natural inhibitors known as tissue inhibitors of MMPs [6]. During angiogenesis, MMPs have an important role in connective tissue remodeling and in the degradation of basement membrane and surrounding ECM [7,8]. Thus, the regulation of angiogenesis may be dependent on the intensity of expression of these enzymes as well as endocrine and growth factors. Any genetic polymorphism in the loci encoding MMPs and their corresponding plasma levels could belong to the risk factors participating in PDR development.

MMPs belong to a family of zinc-containing ECM-degrading enzymes that share common structural and functional properties [9]. The important molecules controlling the formation of new vessels are gelatinase A (MMP-2) and gelatinase B (MMP-9), which have the ability to cleave type IV collagen, a major component of the basement membrane. Some studies reported that both MMPs play an important role in the regulation of angiogenesis and may be relevant to the development of the proliferative phase of DR [10-12].

Genetic variability in the regulatory regions of the genes is one of the significant factors that influence levels of MMPs. Several polymorphisms have been identified in the promoter and coding regions of *MMP-2* and *MMP-9* [13-16]. In this study, we examined five polymorphisms in *MMP-2* (−168G/T, −735C/T, −790T/G, −1306C/T, and –1575G/A) and two polymorphisms in *MMP-9* (−1562C/T and R279Q). We employed the following selection criteria: frequency of the minor allele ≥5%; and previous evidence that sequence variants in the promoters influence expression as well as levels of MMPs. Polymorphism –1306C/T of *MMP-2* was shown to be linked to a strikingly lower promoter activity associated with the T-allele [14]. Similarly, SNP –1562C/T of *MMP-9* was found to influence transcription in an allele-specific manner, the T-allele having a greater strength than the C-allelic promoter [17]. We hypothesized that susceptibility to the development of PDR may be associated with the presence of particular alleles at the MMPs loci.

The aim of the present study was to employ PDR to find a possible association of genetic variations in *MMP-2* and *MMP-9*. Furthermore, we determined MMP-2 and MMP-9 plasma levels in subjects studied and analyzed mutual relationships between plasma level of product and genetic variability within the particular gene.

## Methods

### Subjects

The present study enrolled 490 unrelated Caucasian subjects, 304 of whom had type 2 diabetes mellitus (DM). All participants were divided into three groups: diabetics with PDR (PDR), diabetics without PDR (non-PDR), and nondiabetics (non-DM). DM was diagnosed previously according to the World Health Organization criteria. All diabetic participants were on some form of treatment. Mean known duration of DM was 11.4±0.56 years (mean±SE). The PDR group consisted of 129 patients (66 men and 63 women), and their average age was 62.3±0.72 years (range 47–79 years). All PDR subjects regularly attended a specialized diabetology unit of the Department of Ophthalmology, University Hospital Brno, Czech Republic. PDR was assessed by direct ophthalmoscopy through dilated pupils of PDR patients and classified as proliferative according to the early treatment diabetic retinopathy (ETDR) criteria [18].Ophthalmologic examinations of PDR patients were performed several times per year. The non-PDR group (n=175) was composed of 175 participants (80 men and 95 women) whose average age was 64.3±0.95 years. The non-DM group had 186 participants (61 men and 125 women) who had no personal history or clinical signs of diabetes. The average age of participants in this group was 59.4±0.99 years. Only the non-DM group (control group) had no evidence of cardiovascular disease, cancer, or allergy, and they were not taking any long-term medication. Signed informed consent was obtained from every participant in each group before their insertion in the study. The present study was conducted according to the principles of the Declaration of Helsinki and was approved by the Ethical Committee, Medical Faculty, Masaryk University, Brno, Czech Republic.

### PCR for *MMP-2* and *MMP-9* single nucleotide polymorphisms

Peripheral blood (5 ml) of all investigated subjects was obtained and mixed with EDTA. Genomic DNA was isolated from 5 ml EDTA-anticoagulated peripheral blood leukocytes by a standard extraction method and used as a template for PCR. Reactions were performed in a final volume of 25 µl, containing 50 mM KCl, 10 mM TRIS-HCl buffer, pH 8.4, 1.5 mM MgCl_2_, 10 pmol of each primer (Table 1), 200 µM dNTP, 1 µg of genomic DNA in the presence of 0.7 U of Taq polymerase (MBI Fermentas, Glen Burnie, MD). After the initial denaturation step (95 °C for 5 min), each cycle (of additional 30 cycles) consisted of a 95 °C denaturation for 30 s, 30 s of annealing, a 72 °C extension for 30 s, and a final extension lasting 8 min at 72 °C. Polymorphisms (−735C/T, −790T/G, −1306C/T, and –1575G/A) were genotyped by PCR and subsequent restriction with specific endonucleases according to methods described elsewhere [15]. Those polymorphisms were analyzed by the standard PCR method which was mentioned above. The primer sequences, annealing temperatures, PCR product and locations for all methods are given in Table 1. In the case of the −168G/T polymorphism, 15 μl aliquots of the PCR product were digested with 3 U of BseDI (MBI Fermentas) for 5 h at 55 °C. Digestion revealed fragments of 26 bp, 194 bp, and 205 bp for the wild-type allele and 26 bp and 399 bp for the mutated allele.

**Table 1:** PCR reaction conditions

**Gene**	**SNP**	**Primer sequence (sense/antisense)**	**T_a_ (°C)**	**Product (bp)**
MMP-2				
	−168G/T	5′-CTGACCATTCCTTCCCGTTC-3′	51	425
		5′-CGCCTGAGGAAGTCTGGAT-3′		
	−735C/T	5′-ATAGGGTAAACCTCCCCACATT-3′	59	300
		5′-GGTAAAATGAGGCTGAGACCTG-3′		
	−790T/G	5′-GGGTCTTTGTGACCTCGATC-3′	56	118
		5′-GGTAAAATGAGGCTGAGACCTG-3′		
	−1306C/T	5′-CTTCCTAGGCTGGTCCTTACTGA-3′	49	193
		5′-CTGAGACCTGAAGAGCTAAAGAGCT-3′		
	−1575G/A	5′-ACTGACTCTGGAAAGTCAGAGCAG-3′	60	269
		5′-GGCACAGGGTGAGGGGATGG-3′		
MMP-9				
	−1562C/T	5′-ATGCTCATGCCCGTAATCCT-3′	65	329
		5′-GGGGTAGTATCACTCTGTCACC-3′		
	R279Q	5′-CTCGCCCCAGGACTCTACAC-3′	60	95
		5′-GTGCAGGCGGAGTAGGATT-3′		

The table summarizes the primer sequences, annealing temperatures, and PCR product lengths.

Polymorphisms −1562C/T and R279Q in *MMP-9* were detected using a modified PCR method based on the published single-strand conformation polymorphism technique [16]. Aliquots (10 μl) of the −1562C/T PCR product were digested with 3 U PaeI (MBI Fermentas) for 5 h at 37 °C. Digestion revealed fragments of 144 and 185 bp for the mutated allele. The wild type allele C lacked the PaeI restriction site. In the case of the R279Q polymorphism, 10 μl of PCR product were digested with 3 U of SmaI (MBI Fermentas) for 5 h at 30 °C. Digestion revealed fragments of 22 and 73 bp for the wild type allele. The mutated allele Q lacked the SmaI restriction site.

### Determination of metalloproteinase-2 and metalloproteinase-9 plasma levels

For determination of plasma levels of MMPs the human MMP-2 and MMP-9 ELISA Kits (RayBiotech, Inc., Norcross, GA) were used according to the manufacturer's instructions. The minimum detectable dose was less than 80 pg/ml of MMP-2 and 10 pg/ml of MMP-9, respectively.

### Statistical analysis

Differences in genotype distribution and consistency with Hardy–Weinberg equilibrium were tested by chi-square test. Differences in allele frequencies of SNPs were tested by two-tailed Fisher exact test. PHASE v. 2.0 [19] software was used to resolve a sample of phase-unknown multilocus genotypes and to estimate population haplotype frequencies by the Bayesian-based algorithm. Comparison of the estimated haplotype frequencies was performed as omnibus testing of differences in haplotype frequency profiles between the two groups (statistical significance assessed empirically via permutation testing). In addition, haplotype-specific effects were analyzed using inferred haplotype pairs by computing chi-square statistics.

Normally distributed metric parameters (Kolmogorov–Smirnov test) were presented as mean ± standard error of the mean (SE), others as median (range). Mann–Whitney U-test, Kruskal–Wallis ANOVA and Spearman rank correlation tests were used where appropriate. Values of p<0.05 were considered to be statistically significant. Bonferroni correction (p_corr_) was applied in case of multiple comparisons modifying the significant p-value threshold according to number of comparisons. Statistical analyses were performed using program package Statistica for Windows (StatSoft Inc., Tulsa, OK).

## Results

### Genetic polymorphisms of metalloproteinase-2 and metalloproteinase-9 genes and proliferative diabetic retinopathy

Genotype frequencies of all SNPs (−168G/T, −735C/T, −790T/G, −1306C/T,  –1575G/A, −1562C/T, and R279Q) in the individual groups are shown in Table 2. Genotype distribution of any of the seven polymorphism studied did not differ from Hardy–Weinberg equilibrium in any group (p>0.05). Similarly, genotype frequencies of any polymorphisms did not differ significantly between groups in pair-wise comparison (p>0.05). Comparison of allele frequencies showed statistically significant difference in allele frequencies of the -1306C/T polymorphism between the PDR versus non-DM groups (p=0.024), however, after the correction for multiple comparison the difference did not remained significant (pcorr>0.05). The calculated odds ratio (OR) for the CC and CT genotypes was 1.41 (95% confidence interval (CI): 0.52±3.87).

**Table 2:** Genotype distributions of MMP-2 and MMP-9 polymorphisms

	**MMP-2 −168G/T**					
	GG (%)	GT (%)	TT (%)	(1) vs. (2)	(2) vs. (3)	(1) vs. (3)
PDR (n=128) ^(1)^	106 (83)	19 (15)	3 (2)			
non-PDR (n=175) ^(2)^	151 (86)	23 (13)	1 (1)	NS NS	NS NS	NS NS
non-DM (n=186) ^(3)^	157 (84)	29 (16)	0 (0)			
	**MMP-2 −735C/T**					
	CC (%)	CT (%)	TT (%)	(1) vs. (2)	(2) vs. (3)	(1) vs. (3)
PDR (n=128) ^(1)^	95 (74)	32 (25)	2 (1)			
non-PDR (n=175) ^(2)^	136 (78)	38 (22)	1 (0)	NS NS	NS NS	NS NS
non-DM (n=186) ^(3)^	142 (76)	40 (22)	4 (2)			
	**MMP-2 −790T/G**					
	TT (%)	TG (%)	GG (%)	(1) vs. (2)	(2) vs. (3)	(1) vs. (3)
PDR (n=128) ^(1)^	74 (57)	46 (36)	9 (7)			
non-PDR (n=175) ^(2)^	92 (54)	66 (38)	14 (8)	NS NS	NS NS	NS NS
non-DM (n=186) ^(3)^	90 (48)	85 (46)	11 (6)			
	**MMP-2 −1306C/T**					
	CC (%)	CT (%)	TT (%)	(1) vs. (2)	(2) vs. (3)	(1) vs. (3)
PDR (n=128) ^(1)^	79 (61)	44 (34)	6 (5)			
non-PDR (n=175) ^(2)^	94 (54)	66 (38)	15 (9)	NS NS	NS NS	NS (**p=0.024**)
non-DM (n=186) ^(3)^	90 (48)	84 (45)	12 (7)			
	**MMP-2 −1575G/A**					
	GG (%)	GA (%)	AA (%)	(1) vs. (2)	(2) vs. (3)	(1) vs. (3)
PDR (n=128) ^(1)^	73 (57)	49 (38)	7 (5)			
non-PDR (n=175) ^(2)^	94 (54)	66 (38)	15 (9)	NS NS	NS NS	NS NS
non-DM (n=186) ^(3)^	90 (48)	84 (45)	12 (7)			
	**MMP-9 −1562C/T**					
	CC (%)	CT (%)	TT (%)	(1) vs. (2)	(2) vs. (3)	(1) vs. (3)
PDR (n=128) ^(1)^	94 (73)	32 (25)	3 (2)			
non-PDR (n=175) ^(2)^	126 (72)	47 (27)	2 (1)	NS NS	NS NS	NS NS
non-DM (n=186) ^(3)^	134 (72)	49 (26)	3 (2)			
	**MMP-9 R279Q**					
	RR (%)	RQ (%)	QQ (%)	(1) vs. (2)	(2) vs. (3)	(1) vs. (3)
PDR (n=128) ^(1)^	44 (34)	69 (54)	16 (12)			
non-PDR (n=175) ^(2)^	73 (42)	83 (47)	19 (11)	NS NS	NS NS	NS NS
non-DM (n=186) ^(3)^	70 (38)	86 (46)	30 (16)			

There were no significant differences between the groups when comparing genotype distributions (p>0.05). The statistically significant difference was found in allele frequencies of −1306C/T polymorphism between the proliferative diabetic retinopathy (PDR versus non-diabetes mellitus (DM) groups (p=0.024; p_corr_>0.05). Genotype distribution of any polymorphism did not differ from Hardy-Weinberg equilibrium in any group (p>0.05). The p-values of allele frequencies (two-tail Fisher exact test) are given in parentheses.

Testing for association carried separately for males and females revealed statistically significant gender-specific association of the two *MMP-2* polymorphisms (−168G/T and −1306C/T), in particular, differences in genotype as well as in allele frequencies between the PDR and non-DM groups (Table 3). In female subjects, the significant differences in genotype distribution (p=0.040) and allele frequency (p=0.016) were found for −168G/T polymorphism. Female subjects carrying the −168T allele had a 2.45 fold higher risk of developing PDR (OR=2.45, 95% CI: 1.16–5.20) than those not carrying this allele. On the other hand, the statistically significant difference in genotype distribution (allele frequency) of −1306C/T polymorphism was found in male subjects between the same groups (p=0.039 and p=0.024). The calculated OR for patients carrying the −1306C allele was 1.88 (95% CI: 1.05–3.40). However, after taking the number of comparisons into account, we found none of the significances remained statistically significant (p_corr_>0.05).

**Table 3:** Comparisons of genotype distributions of the two *MMP-2* polymorphisms in male and female subjects.

**Males**	**MMP-2 −168G/T**						
	GG (%)	GT (%)	TT (%)	(1) vs. (2)	(2) vs. (3)	(1) vs. (3)	
PDR (n=65) ^(1)^	57 (88)	7 (11)	1 (1)				
non-PDR (n=80) ^(2)^	69 (86)	11 (14)	0 (0)	NS NS	NS NS	NS NS	
non-DM (n=61) ^(3)^	46 (75)	15 (25)	0 (0)				
	**MMP-2 −1306C/T**					
	CC (%)	CT (%)	TT (%)	(1) vs. (2)	(2) vs. (3)	(1) vs. (3)	
PDR (n=66) ^(1)^	42 (64)	24 (36)	0 (0)				
non-PDR (n=80) ^(2)^	43 (54)	32 (40)	5 (6)	NS NS	NS NS	**p=0.039** (**p=0.024**)	
non-DM (n=61) ^(3)^	29 (47)	28 (46)	4 (7)				
**Females**	**MMP-2 −168G/T**						
	GG (%)	GT (%)	TT (%)	(1) vs. (2)	(2) vs. (3)	(1) vs. (3)	
PDR (n=63) ^(1)^	49 (78)	12 (19)	2 (3)				
non-PDR (n=95) ^(2)^	82 (86)	12 (13)	1 (1)	NS NS	NS NS	**p=0.040** (**p=0.016**)	
non-DM (n=125) ^(3)^	111 (89)	14 (11)	0 (0)				
	**MMP-2 −1306C/T**					
	CC (%)	CT (%)	TT (%)	(1) vs. (2)	(2) vs. (3)	(1) vs. (3)	
PDR (n=63) ^(1)^	37 (59)	20 (32)	6 (9)				
non-PDR (n=95) ^(2)^	51 (54)	34 (36)	10 (10)	NS NS	NS NS	NS NS	
non-DM (n=125) ^(3)^	61 (49)	56 (45)	8 (6)				

The table shows the statistical significance of differences in allele frequencies (the p-values of two-tail Fisher exact test are given in parentheses) and genotype distribution (χ^2^ test) between the studied groups for the two sexes separately. In female subjects, the significant difference was found in allele frequency of the −168G/T polymorphism between the proliferative diabetic retinopathy (PDR) and non-diabetes mellitus (DM) groups (p=0.016, odds ratio (OR)=2.45, 95% CI: 1.16-5.20). In male subjects the −1306C/T polymorphism exhibited statistically significant difference in allele frequency between the same groups (p=0.024, OR=1.88 (95% CI: 1.05-3.40). However, after the correction for multiple comparisons differences were no longer significant (p_corr_>0.05).

Haplotype analysis was performed to compare the sum effect of genetic variability of the four polymorphisms (−1306C/T, −790T/G, −735C/T and −168G/T) in *MMP-2*. The −1575G/A polymorphism was omitted due to the strong linkage disequilibrium between −1306C/T and −1575G/A polymorphisms. Haplotypes were constructed from genotype data of all subjects computationally using PHASE software with the assumption of mutual independence of haplotypes. No statistically significant difference was found in haplotype frequencies between the groups studied (omnibus p>0.05; 10,000 permutations). Analyses performed separately for men and women revealed a statistically significant difference in males (omnibus p=0.042; 10,000 permutations); while no significant difference was observed in females (omnibus p=0.738, 10 000 permutations). The pair-wise comparison of groups in males showed significant differences in haplotype frequencies between PDR versus non-PDR groups (p=0.031). To analyze haplotype-specific effects on the trait (i.e., presence of PDR), we asigned pairs of haplotypes to individuals. Subsequently, numbers of individuals with this particular haplotype were counted. Table 4 shows the absolute numbers of haplotypes in particular groups that were inferred retrospectively with probability more than 90%. Carriers of haplotypes with frequencies <1% in all three groups were pooled together as “rare.” Separate one-degree of freedom tests were conducted for a series of 2×2 contingency tables testing the frequency of each specific haplotype versus all others between particular groups (PDR versus non-PDR and PDR versus non-DM). Two specific haplotypes, - CGCG (OR=7.57, i.e., risk haplotype) and TGCG (OR=0.56, i.e., protective haplotype), exhibited significant associations with PDR in male patients (p=0.030 and p=0.041, respectively), after the correction for multiple tests, however, the significances did not remained significant (p_corr_>0.05).

**Table 4:** Haplotypes derived from *MMP-2* polymorphisms in male patients

**Haplotype**	**PDR (%)**	**non-PDR (%)**	**χ^2^** **(p-value)**	**OR (95% CI)**	**non-DM (%)**	**χ^2^** **(p-value)**	**OR (95% CI)**
CTCG	73 (55)	83 (52)	NS	-	53 (43)	NS	-
TGCG	23 (17)	44 (28)	**0.041**	0.56 (0.32-0.98)	34 (28)	**0.046**	0.55 (0.30-0.99)
CTTG	21 (16)	21 (13)	NS	-	17 (14)	NS	-
CTCT	9 (7)	11 (7)	NS	-	15 (12)	NS	-
CGCG	6 (5)	1 (1)	**0.030**	7.57 (0.89-63.71)*	1 (1)	NS	-
Rare	0 (0)	0 (0)	NS	-	2 (2)	NS	-
PDR vs. non-PDR			0.031**		PDR vs. non-DM	0.068**	

*MMP-2* haplotypes inferred from the four single nucleotide polymorphisms (SNPs; ordered from the 5′ to 3′as follows: −1306C/T, −790T/G, −735C/T, and −168G/T) in males. Haplotypes with frequencies less than 1% in both groups were pooled together as “rare”. χ^2^ (p-value) was derived from single 2x2 contingency tables testing the frequency of each specific haplotype vs. all others between particular groups (proliferative diabetic retinopathy [PDR] versus non-PDR and PDR versus non-diabetes mellitus [DM]). The double asterisk represents omnibus p-value was assessed empirically by permutation testing (PHASE output, 10 000 permutations). The asterisk means an odds ratio>1.5 was significantly associated with PDR.

### Plasma levels of metalloproteinase-2 and metalloproteinase-9 versus genetic variation

MMP-9 plasma levels were 12.3 ng/ml (8.0–16.7 ng/ml) in the PDR group, 11.3 ng/ml (7.9–13.1 ng/ml) in the non-PDR group and 10.3 ng/ml (2.0–13.7 ng/ml) in the non-DM group (p=0.001, Kruskal–Wallis ANOVA). Using the Mann–Whitney U test, we observed significant differences in the plasma levels of MMP-9 between the PDR or non-PDR groups and the non-DM group (p<0.001 and p=0.006, respectively), while we observed no notable difference between the PDR and non-PDR groups (p=0.079). No significant differences were found between *MMP-9* genetic variations (groups defined according to the −1562C/T or R279Q polymorphism genotypes), and plasma levels of the MMP-9 in the whole sample or within each gender and study group separately (p>0.05).

MMP-2 plasma levels were 401.5 ng/ml (142.4–633.4 ng/ml) in the PDR group, 277.9 ng/ml (118.0–473.5 ng/ml) in the non-PDR group, and 260.8 ng/ml (44.8–428.2 ng/ml) in the non-DM group (p<0.001, Kruskal–Wallis ANOVA). The Mann–Whitney U test revealed statistically notable differences in MMP-2 between the PDR and non-PDR or non-DM groups (p=0.001 and p<0.001, respectively). A comparison of plasma levels of MMP-2 corresponding to particular genotypes of *MMP-2* SNPs showed a statistically significant difference for the −1306C/T variant (p=0.011, Kruskal–Wallis ANOVA). The plasma levels were significantly higher in the CC and CT genotypes, compared to TT in diabetic patients (p=0.009, Mann–Whitney U test). No gender-specific differences were ascertained for the −1306C/T variant and *MMP-2*; this was mostly due to incomparably low numbers of TT genotypes after splitting the whole group into two genders.

The comparison of plasma levels of MMP-2 among the haplotype groups–i.e., risk (CGCG) versus the protective (TGCG) versus all others–revealed the highest MMP-2 plasma levels were in the risk haplotype group (p=0.043, Kruskal–Wallis ANOVA). The median (range) of MMP-2 was 388.1 ng/ml (186.4–633.4 ng/ml) in the PDR-risk haplotype, 269.2 ng/ml (185.3 – 473.5 ng/ml) in the protective haplotype, and 259.5 ng/ml (44.8–425.8 ng/ml) in others, respectively. As with the previous situation, gender-specific differences were not ascertained due to low numbers of risk haplotypes after dividing the study population into women and men.

Furthermore, because advanced age and DM duration are risk factors for PDR, we analyzed the correlation between the plasma levels of MMP-2 and MMP-9 and the aforementioned parameters. A statistically significant Spearman rank correlation was found between plasma level of MMP-2 and DM duration (Rs=0.376; p=0.005) while no other correlation was ascertained.

## Discussion

So far, no association study of genetic variability in *MMP-2* and *MMP-9* and PDR has been published. Retinal angiogenesis is a hallmark of PDR, involving the production of angiogenic factors as well as synthesis of ECM proteins. Both *MMP-2* and *MMP-9* are interesting candidate genes for PDR because of their role in connective tissue remodeling and in regulation of extracellular matrix during angiogenesis [20].

Analysis of individual polymorphisms showed that their frequencies in Czech Caucasian subjects are similar to those described previously [15,21], however, none of individual polymorphism was associated with PDR in this study. Allele frequencies of the −168G/T and −1306C/T polymorphisms differed marginally in female and male study participants between the PDR and control groups; however, the interpretation of suggestive gender-specific differences is not straightforward. One possible explanation might be a different modulatory effect of sex hormones on cytokine-regulated gene expression of *MMP-2* or regulation of MMP-2 plasma levels [22,23]. While single-locus association studies have prevailed in the past, haplotype-based association studies offer more robust approach to the analysis of complex traits since these studies consider the whole genetic variability within the particular locus as a unit. Previously we showed that TGF-β_1_ haplotypes might play a role in PDR susceptibility [24]. Furthermore, specific haplotypes based on SNPs in the promoter region of the MMP-2 gene were associated with coronary artery disease [15]. The present study identified two specific haplotypes, CGCG (risk) and TGCG (protective), which were marginally associated with PDR in male patients. This is in agreement with the results of single-locus association since the C allele of the −1306C/T variant significantly associated with PDR in male subjects defines the two haplotypes.

Several previous studies quantified levels of MMP-2 and MMP-9 proteins in PDR subjects [25-27]. Data were obtained by various methods (zymography, immunoblot, and immunohistochemistry), and the results showed increased concentrations of MMP-2 and MMP-9 in patients with PDR [28,29]. Using ELISA, we confirmed that PDR is associated with the highest plasma levels of both MMP-2 and MMP-9 compared to non-DM as well as non-PDR groups. Effects of genetic variability on the level of gene expression of MMPs were also described by Price and Zhang [14,17]. The transition C→T of the −1306C/T polymorphism displayed a lower promoter activity for the T allele [17]. Furthermore, the −1575G allele increased transcription activity and had an independent additive effect with the −1306C allele [30]. Our results support an importance of the −1306C/T polymorphism as a factor determining the intermediate phenotype, since the risk CC/CT genotypes and risk haplotype containing the C allele exhibited significantly highest plasma level of MMP-2 protein. Unfortunately, we could not pursue the possible effect of male gender-specific association in the study of its effect on MMP-2 levels since the numbers were too low in some groups of genotypes or haplotypes to allow for an adequate comparison. Nevertheless, the topic is intriguing enough to warrant further study on a larger group.

In conclusion, we demonstrated plasma levels of MMP-2 are notably higher in patients with PDR, and they exhibit significant −1306C/T genotype- and haplotype-specific differences with higher levels in the CC/CT genotypes and CGCG haplotype. Although we have not been able to prove statistically significant association of those *MMP-2* gene variants with PDR, both suggestive risk genotypes and haplotype are overrepresented in the PDR group which explains the observed difference in the MMP-2 plasma levels between patient groups. The gender-specific effects on *MMP-2* regulation require further study.
